# Serial Systemic *Candida albicans* Infection Highlighted by Proteomics

**DOI:** 10.3389/fcimb.2019.00230

**Published:** 2019-06-26

**Authors:** Glaucia Sayuri Arita, Jean Eduardo Meneguello, Karina Mayumi Sakita, Daniella Renata Faria, Eduardo Jorge Pilau, Luciana Dias Ghiraldi-Lopes, Paula Aline Zanetti Campanerut-Sá, Érika Seki Kioshima, Patrícia de Souza Bonfim-Mendonça, Terezinha Inez Estivalet Svidzinski

**Affiliations:** ^1^Department of Clinical Analysis and Biomedicine, State University of Maringá, Maringá, Brazil; ^2^Department of Chemistry, State University of Maringá, Maringá, Brazil

**Keywords:** *Candida albicans*, systemic candidiasis, serial passage, virulence, host–pathogen interaction, proteomics, mass spectrometry, LC-MS/MS

## Abstract

*Candida albicans* is the major pathogen isolated from nosocomial bloodstream infections, leading to higher mortality rates. Thus, due to its clinical relevance, studies aiming to understand host–pathogen interactions in *C. albicans* infection are necessary. Therefore, we performed proteomic analysis using a murine model of serial systemic infection by *C. albicans* to evaluate possible changes in the protein profile of the pathogen over time. Firstly, we observed a reduction in the median survival time of infected animals with increasing passage number, suggesting a higher pathogenicity acquired during repeated infections. By LC-MS/MS, it was possible to obtain protein profiles from the wild-type strain (WT) and compare them to proteins extracted from *Candida* cells recovered from infected tissues during passages one, three, and four (P1, P3, and P4). We obtained 56, 29, and 97 proteins in P1, P3, P4, respectively, all varying in abundance. Regarding biological processes, the majority of proteins were related to carbohydrate metabolism, stress responses and amino acid metabolism. The proteins were also categorized according to their potential role in virulence traits, such as biofilm production, yeast-to-hyphae transition, phenotypic switching, proteins related to stress responses, and uncharacterized proteins. Therefore, serial infection in combination with proteomic approach enabled us to deepen the existing knowledge about host-pathogen interactions.

## Introduction

*Candida* spp. is one of the most common agents of nosocomial bloodstream infections worldwide, leading to high morbidity and mortality rates. Among them, *C. albicans* is still the major pathogen isolated (Lamoth et al., 2018). Some of the main risk factors associated with candidemia are the use of antimicrobial agents, corticosteroid usage, chemotherapy, hematological malignancies, central venous catheter usage, gastrointestinal surgery, parenteral nutrition, and hospitalization in the Intensive Care Unit (ICU), which facilitates dissemination of *Candida* among patients (Pfaller and Diekema, 2007). The risk of cross-transmission between healthcare workers' hands and ICU surfaces by strains with greater expression of virulence factors has also been previously described (Sakita et al., 2017).

Previous studies demonstrated that a model of serial infection provided insights into host–pathogen adaptations. After serial passages through the kidney, strains were recovered with higher phenotypic variability (Lüttich et al., 2013); however, no overall virulence trend was observed. On the other hand, other authors recovered a mutant defective in oxidative phosphorylation, but it was more resistant to phagocytosis and killing by neutrophils and macrophages after the fifth passage, demonstrating how *C. albicans* behaved in the infection (Cheng et al., 2007).

During interaction with the host, *C. albicans* faces different pH, osmolarity, nutritional starvation, and oxidative stress conditions, then responds to them by modifying its transcriptional profile to adapt to the surrounding environment (Calderone and Fonzi, 2001; Hube, 2004; Li et al., 2015). In the same way, the protein profile also correlates with these changes, although some discrepancies between transcriptional and proteomic profiles exist (Lee et al., 2011). Therefore, proteomics is a valuable tool to understand adaptations that occur in *C. albicans* to evade host defenses and to invade tissues, contributing to infection. Thus, this is the first study that aimed to use a proteomic view to elucidate host–pathogen interactions after serial systemic candidiasis in a murine model.

## Methods

### Ethics Statement

All procedures were approved by the Ethics Committee for Animal Use of the State University of Maringá, PR, Brazil, under the protocol number CEUA 7261020418 which is in accordance with Brazil's National Council for the Control of Animal Experimentation (CONCEA).

### Strain and Culture Conditions

The reference strain *C. albicans* SC5314 was used for the initial infection in a murine model of disseminated candidiasis. First, it was cultured on Sabouraud Dextrose Broth (Difco). Next, a loop was streaked on CHROMagar™ *Candida* (Becton Dickinson). Then, a single colony was streaked on a Sabouraud Dextrose Agar (SDA) (Difco) plate and incubated for 24 h at 35°C. An inoculum of 3.5 × 10^5^ yeast cells was prepared in phosphate buffered saline (PBS, pH 7.4).

### Murine Model of Serial Disseminated Candidiasis and Survival Curve

**Figure 1:**
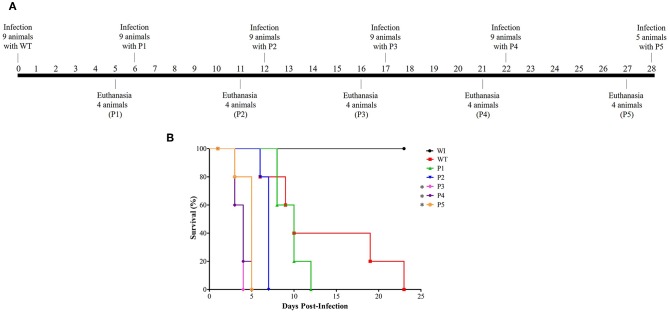
Model of how serial systemic candidiasis reduces the survival time of animals infected by yeasts recovered from passages. A total of 52 animals were used throughout the experiment for each biological replicate. **(A)** Serial infection timeline starting with nine mice being infected by wild-type *C. albicans* SC5314 strain (WT) at day 0, followed by euthanasia of four animals at day 5 to recover their colonies to use for inoculum preparation to infect nine animals for the next passage. The remaining five animals were used to construct the survival curve. A timeline was designed from passage 1 to 5 (P1–P5). **(B)** Survival curve from a group of five animals each infected with 3.5 × 10^5^ cells of *C. albicans* and monitored until their natural death. In addition, healthy mice (without infection; WI, *n* = 2) were also followed up. This survival curve represents at least two independent experiments. A log-rank test for multiple comparisons between each passage and the control was applied; *p = 0.0031 (P3), *p = 0.0019 (P4), and *p = 0.0035 (P5).

Serial systemic candidiasis consisted of initially infect nine female Balb/c mice with wild-type *C. albicans* SC5314 (WT) with 0.1 ml of prepared inoculum via the lateral tail vein. A total of 52 animals were used throughout the experiment for each biological replicate as shown in Figure 1: 9 animals for each passage (infection with WT and infection with colonies recovered from passage P1–P4), 5 mice infected with colonies recovered from P5 for survival curve only, and 2 animals without infection (WI). Thus, considering the non-homogenous death rate, we divided the mice into two groups. One group of five animals was used for the survival curve experiment. The animals were monitored through their behavior parameters, weighed daily, and followed up until their deaths.

The other group of four animals was humanely euthanized 5 days post-infection. The kidney was aseptically removed, homogenized in lysis buffer (200 mM NaCl, 5 mM EDTA, 10 mM Tris, 10% glycerol v/v, pH 8.30), plated on SDA, and incubated for 24 h at 35°C. Colonies recovered from the infected kidney were used to prepare the inoculum for the subsequent infection as described for WT. This scheme was performed until colonies were obtained from the fifth passage, totaling five serial passages (P1–P5). Healthy animals (without infection) were humanely euthanized at the end of the experiment and were used as reference in the survival curve and for monitoring the animals' weight.

### Proteomics

For proteome analysis, *Candida* cells recovered from kidney and plated on SDA were collected and centrifuged for 5 min at 3,000 rpm. The pellet was washed three times with ultrapure water and resuspended in 1,100 μl of buffer containing 7 M urea, 2 M thiourea, and 40 mM DTT (Fiorini et al., 2016). Then, the suspension was sonicated (30 bursts of 10 s ON, 30 s OFF at 100% amplitude). After that, the lysate was centrifuged at 4,800 rpm for 10 min at 4°C, and the supernatant was collected and stored at −80°C. The Bradford assay was performed for protein quantification, using bovine serum albumin (Sigma) as a standard. Prior to digestion, proteins were denatured with 8 M urea, then reduced with 5 mM dithiothreitol (DTT) and alkylated with 14 mM iodoacetamide (IAA). To quench the remaining IAA, 5 mM DTT was added. Samples were diluted with four volumes of 50 mM ammonium bicarbonate, and 1 mM calcium chloride was added, followed by trypsin (V5280–Promega) digestion at 37°C overnight (ratio of 1 μg enzyme to 100 μg protein). Then, samples were acidified with 0.4% trifluoroacetic acid to inactivate the trypsin and desalted using SepPak tC18 cartridges (WAT 036820).

Samples were analyzed by LC-MS/MS using ultra-high-performance liquid chromatography (Shimadzu, Nexera X2, Japan) coupled to high-resolution mass spectrometry (Impact II, Bruker Daltonics Corporation, Germany) and equipped with an electrospray ionization source. Ten microliters of the sample were loaded at a flow rate of 0.1000 ml/min. The capillary voltage was operated in positive ionization mode, set at 4,500 V and with an end plate offset potential of −500 V. The dry gas parameters were set to 7.0 L min^−1^ at 180°C with a nebulization gas pressure of 2.0 bar. Data were collected from m/z 150–2,200 with an acquisition rate of five spectrums per second, and the ions of interest were selected by auto MS/MS scan fragmentation. Chromatographic separation was performed using gradient A containing purified water and 0.1% formic acid (v/v) and B containing acetonitrile with 0.1% formic acid (v/v) using a C18 column (Shim-pack GIST−3 μm × 1.0 mm × 250 mm). The gradient used was 2, 35, 95, 95, and 2% of B in 2, 60, 90, 110, and 111 min, respectively, and stopped at 120 min.

Files obtained by the mass spectrometer were normalized using Data Analysis software (Bruker, Germany), converted in a vendor-free file by MSFile from the ProteoWizard Platform (Chambers et al., 2012) and processed in MaxQuant software (version 1.6.2.3) (Tyanova et al., 2015) for label-free quantification (LFQ). The proteins were searched against the reference proteome from *C. albicans* (strain SC5314/ATCC MYA-2876) taxonomy code 237561 from the UniProt Database. Parameters were set for the digestion mode specific for trypsin with a maximum of two missed cleavage sites. Variable modifications were oxidation (M) and acetyl (Protein N-term), and the fixed modification was carbamidomethyl (C) with a maximum of five modifications. False-discovery rates (FDR) were set to 0.01 for both peptide and protein levels. Then, the MaxQuant output file was imported in Perseus (version 1.6.1.3) (Tyanova et al., 2016) for statistical analysis. First, the protein dataset was filtered, removing proteins into categorical parameters: contaminant, only identified by site, and reverse database. For statistical analysis, the level of stringency was set as protein identification with LFQ values >0 in two of the replicates. Thus, data analysis was performed using a two-sample test, considering significant those proteins with –log_10_ (*p* < 0.05) associated with the highest difference (passage vs. WT). The mass spectrometry proteomics data have been deposited to the ProteomeXchange Consortium via the PRIDE (Perez-Riverol et al., 2019) partner repository with the dataset identifier PXD012944.

All proteins were classified according to molecular functions and/or biological processes obtained from UniProt (https://www.uniprot.org/) and the *Candida* Genome Database (http://www.candidagenome.org/). Furthermore, proteins were separated according to their role in virulence factors expressed in *C. albicans*. Biological duplicates were performed for proteomic assay.

### Statistical Analysis

Survival curve data were analyzed by GraphPad Prism version 6.00 for Windows (GraphPad Software, San Diego, CA, USA). Thus, two groups were compared at a time, each passage with control, so the significance was adjusted to account for multiple comparisons. In this way, log-rank (Mantel-Cox) test was used, and *p*-value of lower than 0.01 was considered statistically significant.

## Results

### Reduction of Survival Time After Serial Systemic Candidiasis

The first step was to evaluate if serial infection, as represented in Figure 1A, could influence survival time in a murine model of systemic candidiasis. We inoculated five mice per passage with 3.5 × 10^5^ *C. albicans* cells and followed up until their death. In Figure 1B, a decreasing trend in the median survival time was observed in animals infected with colonies recovered (*p* < 0.0002 in log-rank test) in a successive manner. After adjusting the significance for multiple comparisons between each passage and the WT, the median survival time was 10 days for the animals infected with the WT and P1 and 7 days for those infected with P2 (*p* = 0.3728 and *p* = 0.0472, respectively). From P3 onwards, a significant reduction of median survival time in relation to the WT was observed [(*p* = 0.0031 (P3), *p* = 0.0019 (P4), and *p* = 0.0035 (P5)], but there were no significant differences among them.

In general, all animals infected lost body weight; furthermore, they became severely ill after the third passage. The group of animals infected with the WT lost 29% of their body weight. Similarly, the groups infected with P1 and P2 lost 22 and 29% of their body weight, respectively, while those infected with P3 and P4 lost 24%. Lastly, animals infected with P5 lost 25% of their body weight. In contrast, healthy mice (without infection; WI) were followed up throughout the experiment, and they presented a weight gain of ~6%. During serial infection, animals presented abdominal contortion and piloerection, and these behavioral parameters were more evident in mice infected with P3 to P5.

### Proteomics Profile After Serial Infection Reveals Proteins Related to Virulence Factors

**Figure 2:**
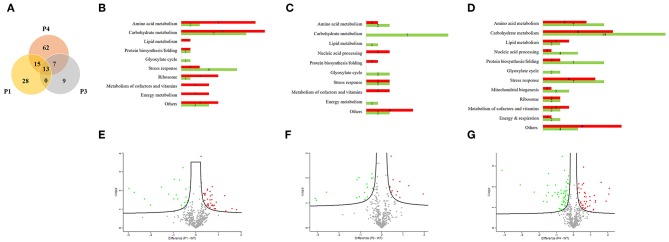
Proteomic analysis from *Candida albicans* recovered from serial systemic infection. **(A)** Venn diagram showing proteins from each passage: 13 proteins are common to all evaluated passages (P1, P3, and P4), 15 proteins are common to P1 and P4, and 7 proteins are common to P3 and P4. The unshared part represents unique proteins: 28, 9, and 62 proteins for P1, P3, and P4, respectively. **(B–D)** Proteins were classified according to their biological process and divided between lower abundance (green bar) and higher abundance (red bar) for P1, P3, and P4. **(E–G)** Volcano plot represents graphically the data from negative logarithmic *P*-values of the *t*-test against the difference in protein intensities between passages and the wild type strain. Proteins with lower abundance are represented by green dots, those with higher abundance by red dots, and those without significant differences by gray dots.

We used a proteomic view to evaluate the protein profile in the most prominent passages (P1, P3, and P4). Therefore, we selected three passages for proteomic analysis plus the WT. We chose P1 because it is the beginning of serial infection and also P3 and P4 because of the significant reduction in survival time. A total of 479 proteins were identified and quantified, and all proteins from passages were compared with respect to their abundance in relation to the WT (Supplementary Material). Thus, a Venn diagram was constructed and showed 13 proteins that changed their abundance in all three passages (Figure 2A). From 56 proteins with different abundance in P1, 15 were in common with P4. From 29 proteins that were different in abundance in P3, 7 were in common with P4. The unique proteins that were significantly different only in one passage compared to WT were 28, 9, and 62 proteins for P1, P3, and P4, respectively. The remaining proteins were not significantly different in any passages in relation to the WT.

We further classified the proteins according to their biological processes and divided them into lower in abundance (green bar) and higher in abundance (red bar) for P1, P3, and P4 (Figures 2B–D). The majority of proteins were related to carbohydrate metabolism in all passages, followed by stress response proteins and amino acid metabolism. We also found proteins involved in protein biosynthesis/folding, lipid metabolism, metabolism of cofactors and vitamins, the glyoxylate cycle and energy metabolism during all passages. Proteins related to mitochondrial biogenesis were found only in P4. Furthermore, from 56 proteins in P1, 35 proteins increased in abundance and 21 decreased in abundance than WT. From 29 proteins in P3, 12 were higher in abundance and 17 lower in abundance when compared with the WT. In the same way, from 97 proteins in P4, 41 were increased in abundance and 56 were decreased in abundance relative to the WT.

A volcano plot was constructed by plotting logarithmic ratios of the protein intensities on the y-axis and negative logarithmic *P*-values of the *t*-test performed from biological duplicate experiments on the x-axis. Proteins that increased in abundance in the passages are represented by red dots, those that decreased in abundance in the passages by green dots, and those without significant differences by gray dots (Figures 2E–G).

In addition to the reduction in survival time of the mice infected with colonies recovered from serial infection, many differentially expressed proteins were obtained from P1, P3, and P4. Thus, in order to associate these findings, we categorized the proteins found in our study in terms of their role in virulence factors, such as biofilm, filamentation, phenotypic switching, stress response, and other (Table 1).

**Table 1:** Proteins categorized according to their role in virulence factors.

	**Gene**	**Protein**	**P1xWT**	**P3xWT**	**P4xWT**	**References**
Biofilm	*CYS3*	Cystathionine gamma-lyase	0.8688	0.6811	0.9481	Gárcia-Sánchez et al., 2004
	*YWP1*	Yeast-form wall Protein 1	0.5545	1.2769	1.6142	Gow et al., 2002; Granger et al., 2005
	*CEF3/EFT3*	Elongation factor 3	0.4013	–	–	Marchais et al., 2005
	*MIR1*	Mir1p	0.4497	–	–	Marchais et al., 2005
	*IFD6*	Ifd6p	1.2875			Ying et al., 2010
	*ADH1*	Adh1p	–	–	0.8220	Thomas et al., 2006
	*CDC19*	Pyruvate kinase	–	–	0.5644	Thomas et al., 2006
	*RHR2*	Glycerol-1-phosphatase	–	–	0.7219	Desai et al., 2015
	*MPG1*	Mannose-1-phosphate guanyltransferase	–	–	0.3982	Marchais et al., 2005
	*MLS1*	Malate synthase	−2.7376	−3.0497	−3.0866	Gárcia-Sánchez et al., 2004
Filamentation	*CYS3*	Cystathionine gamma-lyase	0.8688	0.6811	0.9481	Rashki et al., 2012
	*NOP1*	rRNA methyltransferase	0.4075	–	–	Rashki et al., 2012
	*NOP58*	Nucleolar protein 58	0.3969	–	–	Rashki et al., 2012
	*SIK1*	snoRNP complex protein	0.2416	–	−0.2836	Rashki et al., 2012
	*TIF34*	Eukaryotic translation initiation factor 3 subunit I	–	0.4610	–	Rashki et al., 2012
	*DDR48*	Stress protein DDR48	–	–	1.2947	Dib et al., 2008; Cleary et al., 2012
	*RHR2*	Glycerol-1-phosphatase	–	–	0.7219	Aoki et al., 2013a
	*ACH1*	Acetyl-CoA hydrolase	−0.7261	−0.6624	−1.0314	Rashki et al., 2012
	*CIT1*	Citrate synthase	−0.8699	−1.0656	−1.2239	Rashki et al., 2012
	*SOD1*	Superoxide dismutase [Cu-Zn]	−2.2817	−2.5826	−1.7312	Rashki et al., 2012
	*IFE2*	Ife2p	−1.2083	−1.0490	−1.4998	Rashki et al., 2012
	*AAT21*	Aspartate aminotransferase	−0.5720	–	−0.5414	Rashki et al., 2012
	*MDH1*	Malate dehydrogenase, cytoplasmic	−2.0160	–	−1.5697	Rashki et al., 2012
	*GDH3*	Glutamate dehydrogenase	–	−0.6970	–	Rashki et al., 2012
	*GSY1*	Glycogen [starch] synthase	–	–	−0.2303	Rashki et al., 2012
Phenotypic switching	*OSM2*	Fumarate reductase	0.7646	–	0.5936	Si et al., 2013
	*WH11*	Wh11p	–	−0.8011	–	Huang et al., 2009
Stress response	*SOD1*	Superoxide dismutase [Cu-Zn]	−2.2817	−2.5826	−1.7312	Chauhan et al., 2006; Li et al., 2015
	*SOD3*	Superoxide dismutase	1.6093	1.9415	2.0571	Rashki et al., 2012
	*YHB1*	Flavohemoprotein	0.6299	0.5347	0.3077	Hromatka et al., 2005
	*BLP1*	Blood-induced peptide 1	–	–	1.2168	Aoki et al., 2013b
Other	orf19.36.1	Uncharacterized	−0.6240	–	1.8996	Uncharacterized

We detected several proteins related to biofilm found in the literature. Among them, cystathionine gamma-lyase (Cys3), a protein from amino acid metabolism, was higher in abundance in all passages than in the WT. In contrast, malate synthase (Mls1), which is a key glyoxylate cycle enzyme, was decreased in abundance in all passages (Gárcia-Sánchez et al., 2004). Another protein presented in increased in abundance in all passages was yeast-form wall protein 1 (Ywp1). We also obtained several proteins that were increased in abundance only in P1, such as elongation factor 3 (Cef3), Mir1p, and Ifd6p, and others that were increased only in P4, such as Adh1, pyruvate kinase (Cdc19), glycerol-1-phosphatase (Rhr2), and mannose-1-phosphate guanyltransferase (Mpg1).

In relation to proteins associated with yeast-to hyphae transition, Cys3 was increased in abundance in all passages. There were also proteins that increased in abundance from box C/D snoRNO complex (Nop1, Nop58, and Sik1) in P1 (Rashki et al., 2012). Two other proteins, stress protein DDR48 (Ddr48p) and glycerol 3-phosphatase (Rhr2), were only increased in abundance in P4. Many proteins, such as acetyl-CoA hydrolase (Ach1), citrate synthase (Cit1), superoxide dismutase (Sod1) and Ife2p, were decreased in abundance in all passages. In addition, aspartate aminotransferase (Aat21) and malate dehydrogenase (Mdh1) were decreased in abundance in P1 and P4. Glutamate dehydrogenase (Gdh3) was decreased only in P3 and glycogen synthase (Gsy1) only in P4.

We found proteins that indicate the process of phenotypic switching related to yeast-to-hyphae transition (Si et al., 2013). We found increased abundance of fumarate reductase (Osm2) in P1 and P4 and decreased abundance of Wh11p in P3.

Regarding proteins involved in stress response, superoxide dismutase 1 and 3 (SOD1 and SOD3) were decreased and increased in abundance, respectively, in all three passages. There was also an increased abundance of flavohemoprotein (Yhb1) in all passages and with increased abundance of blood-induced peptide 1 (Blp1) only in P4.

We also detected some proteins that have not yet been characterized, such as orf19.36.1. It is worth noting that this protein is decreased in abundance in P1 but increased in abundance in P4.

## Discussion

*C. albicans* is one of the major pathogens isolated from fungal infections (Lamoth et al., 2018). Once *C. albicans* enters the bloodstream, it can invade many organs and faces different conditions (pH, levels of oxygen and carbon dioxide, nutrients) that will induce *Candida* cells to adapt and cause disease (Calderone and Fonzi, 2001; Hube, 2004; Li et al., 2015). In addition, *C. albicans* expresses virulence factors that will contribute to pathogenicity.

Disseminated systemic candidiasis is related to two main factors: host susceptibility and/or increased fungal virulence during the infectious process (Calderone and Fonzi, 2001; Pfaller and Diekema, 2007). Our results regarding the reduction of survival time of infected animals, in addition to weight loss and worse behavioral parameters, suggest the hypothesis that after serial contact with the host, there was increased virulence in yeasts.

Proteome analysis is an important approach for comprehensive characterization of dynamic variations that occur during adaptation of microorganisms under different conditions. Considering that the metabolic study of microorganism is complex, this technique provides the opportunity to identify proteins that may be targets in *C. albicans* pathogenicity (Lee et al., 2011; Aoki et al., 2013b). To the best of our knowledge, this is the first study to assess the proteomic profile of *C. albicans* after serial passage in a systemic candidiasis model.

Analysis of the biological processes of the proteins identified revealed a more active metabolism with proteins from amino acid and carbohydrate metabolism increased in abundance in P1 when compared with the remaining passages, as seen in P3, which exhibited only proteins decreased in abundance. This fact could possibly be related to adaptation of the pathogen in the host and to the expression of virulence attributes.

Among proteins related to biofilm, Gárcia-Sánchez et al. (2004) described that genes involved in synthesis of sulfur amino acids, such as Cys3, were overexpressed in biofilm, and genes related to glucose repression, such as *MLS1*, were underexpressed, corroborating our results. Furthermore, we identified Ywp1, a glycoprotein of the yeast form cell wall and literature reports that strains lacking this protein show higher adhesivity and biofilm formation. In this way, the elevated abundance of Ywp1 in the three passages compared with the WT implies lower adhesiveness and could be related to greater ability to disseminate during infection in the host (Gow et al., 2002; Granger et al., 2005). All other proteins (Cef3, Mir1, Ifd6, Adh1, Cdc19, Rhr2, Mpg1) related to biofilm were higher in abundance in the passages than in the WT, which is in agreement with the literature (Marchais et al., 2005; Thomas et al., 2006; Ying et al., 2010; Desai et al., 2015). It should be noted that P1 and P4 presented more proteins with significant differences in abundance than P3.

The morphological transition from yeast to hyphae is an important step for infection as mutant strains in hyphal formation demonstrated an attenuated virulence in a murine infection model (Lo et al., 1997). Rashki et al. conducted a study where *C. albicans* morphogenesis was induced in Lee medium at 37°C, and the RNA was extracted for the analysis of gene expression during the early stage of filamentation. They found increased expression of genes from the box C/D snoRNO complex (*NOP1, NOP58*, and *SIK1*), which coincides with our findings, as proteins from the box C/D snoRNO complex were in greater abundance in P1, confirming involvement of ribosome biogenesis during infection (Rashki et al., 2012). Aoki et al. (2013a) identified 16 unique proteins expressed during the transition to hypha in the entire period studied, such as Rhr2. Therefore, in our study, the increased abundance of Rhr2 only in P4 reflects a possible filamentation role during this passage. Furthermore, the presence of Cys3 in all passages, which is involved in sulfur metabolism, could have a role in the yeast-to-hypha transition (Rashki et al., 2012). These authors also found genes that were upregulated (*TIF34* and *TIM9*) and some were downregulated (*ACH1, CIT1, SOD1, IFE2, AAT21, MDH1, GDH3*, and *GSY1*), which can be correlated with our proteomic findings. Therefore, the presence of proteins related to filamentation suggests that transition to hypha form could have contributed to the reduction in survival time of the animals with successive passages.

According to Si et al. (2013), the *OSM2* gene was induced only in opaque filaments, although the white type has been shown to be more virulent in systemic models, and our findings of increased abundance of the corresponding protein Osm2 indicate that after serial infection, a process of phenotypic switching could be occurring. Furthermore, levels of CO_2_ induced white-to-opaque switch, downregulating white-specific genes, such as *WH11* and *EFG1* and upregulating opaque-specific genes, such as *OP4* and *WOR1*, which are important for mating (Huang et al., 2009). Thus, the decreased abundance of Wh11p found in P3 would imply a possible role in virulence for the opaque type as well.

*C. albicans* has six different superoxide dismutase enzymes. Among them, SOD1 is a cytosolic protein dependent on copper-zinc (Cu/Zn) as a cofactor, and SOD3 is dependent on manganese (Mn). Under Cu starvation conditions, such as during *C. albicans* systemic infection in the kidney, there is a switch expression of *SOD1* to *SOD3* controlled by the Cu-sensing regulator Mac1 (Chauhan et al., 2006; Li et al., 2015). This finding is in agreement with our results, denoting low concentrations of Cu during systemic infection.

Host defense can be exercised by macrophages producing reactive oxygen and nitrogen species (Vázquez-Torres and Balish, 1997). Thus, exposure to nitric oxide (NO) has already been shown to induce expression of *YHB1* and *DDR48* in *C. albicans* (Hromatka et al., 2005). These findings are correlated to increased abundance of the respective proteins Yhb1 and Ddr48p during passages. Moreover, *DDR48* has already been described to have a critical role during filamentation and virulence, although it is not a consensus in the literature (Dib et al., 2008; Cleary et al., 2012).

Aoki et al. (2013b) performed quantitative time-course proteomics from *C. albicans* during adaptation to fetal bovine serum (FBS) and found a protein Blp1 that showed tolerance to various stress conditions, revealing its potential role as a virulence factor. Thus, its significant abundance in P4 might reflect acquired tolerance after serial passaging.

We found an uncharacterized protein that was increased in abundance in P4 and whose passage demonstrated significant reduction in animal survival and exhibited more proteins associated with stress responses. Therefore, this protein could be related to a virulence attribute that should be further investigated.

Altogether, our results demonstrated a significant reduction in median survival time with successive passages. Expression of proteins related to virulence factors might have influenced the increased pathogenicity observed. In this way, phenotypic assays highlighted by proteomics findings should be performed to deepen the study of these proteins, focusing on their roles as virulence markers and therapeutic targets.

## Data Availability

The datasets analyzed for this study can be found in the ProteomeXchange Consortium with identifier PXD012944.

## Author Contributions

GA, KS, and DF performed the experiments with animals. GA and JM performed the proteomics experiments. GA, PB-M, and TS wrote the paper. GA, JM, KS, DF, EP, LG-L, PC-S, EK, PB-M and TS analyzed the data and approved the manuscript for submission.

### Conflict of Interest Statement

The authors declare that the research was conducted in the absence of any commercial or financial relationships that could be construed as a potential conflict of interest.

## References

[B1] Aoki W. Tatsukami Y. Kitahara N. Matsui K. Morisaka H. Kuroda K.. (2013b). Elucidation of potentially virulent factors of *Candida albicans* during serum adaptation by using quantitative time-course proteomics. J. Proteomics 91, 417–429. 10.1016/j.jprot.2013.07.031 23948566

[B2] Aoki W. Ueda T. Tatsukami Y. Kitahara N. Morisaka H. Kuroda K.. (2013a). Time-course proteomic profile of *Candida albicans* during adaptation to a fetal serum. Pathog. Dis. 67, 67–75. 10.1111/2049-632X.12003 23620121

[B3] Calderone R. A. Fonzi W. A. (2001). Virulence factors of *Candida albicans*. Trends Microbiol. 9, 327–335. 10.1016/S0966-842X(01)02094-7 11435107

[B4] Chambers M. C. Maclean B. Burke R. Amodei D. Ruderman D. L. Neumann S.. (2012). A Cross-platform toolkit for mass spectrometry and proteomics. Nat. Biotechnol. 30, 918–920. 10.1038/nbt.2377 23051804 PMC3471674

[B5] Chauhan N. Latge J. P. Calderone R. (2006). Signalling and oxidant adaptation in *Candida albicans* and *Aspergillus fumigatus*. Nat. Rev. Microbiol. 4, 435–444. 10.1038/nrmicro1426 16710324

[B6] Cheng S. Clancy C. J. Zhang Z. Hao B. Wang W. Iczkowski K. A.. (2007). Uncoupling of oxidative phosphorylation enables *Candida albicans* to resist killing by phagocytes and persist in tissue. Cell. Microbiol. 9, 492–502. 10.1111/j.1462-5822.2006.00805.x 16987332

[B7] Cleary I. A. MacGregor N. B. Saville S. P. Thomas D. P. (2012). Investigating the function of Ddr48p in *Candida albicans*. Eukaryot. Cell 11, 718–724. 10.1128/EC.00107-12 22523369 PMC3370460

[B8] Desai J. V. Cheng S. Ying T. Nguyen M. H. Clancy C. J. Lanni F.. (2015). Coordination of *Candida albicans* invasion and infection functions by phosphoglycerol phosphatase Rhr2. Pathogens 4, 573–589. 10.3390/pathogens4030573 26213976 PMC4584273

[B9] Dib L. Hayek P. Sadek H. Beyrouthy B. Khalaf R. A. (2008). The *Candida albicans* Ddr48 protein is essential for filamentation, stress response, and confers partial antifungal drug resistance. Med. Sci. Monit. 14, BR113–121. 18509269

[B10] Fiorini A. Rosado F. R. Bettega E. M. S. Melo K. C. S. Kukolj C. Bonfim-Mendonça P. S. (2016). *Candida albicans* protein profile changes in response to the butanolic extract of *Sapindus saponaria* L. Rev. Inst. Med. Trop. Sao Paulo 58:25 10.1590/S1678-9946201658025 27074319 PMC4826078

[B11] Gárcia-Sánchez S. Aubert S. Iraqui I. Janbon G. Ghigo JM. d'Enfert C. (2004). Candida albicans biofilms: a developmental state associated with specific and stable gene expression patterns. Eukaryot. Cell 3, 536–545. 10.1128/EC.3.2.536-545.2004 15075282 PMC387656

[B12] Gow N. A. Brown A. J. P. Odds F. C. (2002). Fungal morphogenesis and host invasion. Curr Opin Microbiol. 5, 366–371. 10.1016/S1369-5274(02)00338-7 12160854

[B13] Granger B. L. Flenniken M. L. Davis D. A. Mitchell A. P. Cutler J. E. (2005). Yeast wall protein 1 of *Candida albicans*. Microbiology 151, 1631–1644. 10.1099/mic.0.27663-0 15870471

[B14] Hromatka B. S. Noble S. M. Johnson A. D. (2005). Transcriptional response of *Candida albicans* to nitric oxide and the role of the *YHB1* gene in nitrosative stress and virulence. Mol. Biol. Cell 16, 4814–4826. 10.1091/mbc.e05-05-0435 16030247 PMC1237085

[B15] Huang G. Srikantha T. Sahni N. Yi S. Soll D. R. (2009). CO_2_ regulates white-to-opaque switching in *Candida albicans*. Curr. Biol. 19, 330–334. 10.1016/j.cub.2009.01.018 19200725 PMC2667266

[B16] Hube B. (2004). From commensal to pathogen: stage- and tissue-specific gene expression of Candida albicans. Curr. Opin. Microbiol. 7, 336–341. 10.1016/j.mib.2004.06.003 15288621

[B17] Lamoth F. Lockhart S. R. Berkow E. Calandra T. (2018). Changes in the epidemiological landscape of invasive candidiasis. J. Antimicrob. Chemother. 73, i4–i13. 10.1093/jac/dkx444 29304207 PMC11931512

[B18] Lee M. V. Topper S. E. Hubler S. L. Hose J. Wenger C. D. Coon J. J.. (2011). A dynamic model of proteome changes reveals new roles for transcript alteration in yeast. Mol. Syst. Biol. 7:514. 10.1038/msb.2011.48 21772262 PMC3159980

[B19] Li C. X. Gleason J. E. Zhang S. X. Bruno V. M. Cormack B. P. Culotta V. C. (2015). *Candida albicans* adapts to host copper during infection by swapping metal cofactors for superoxide dismutase. Proc. Natl. Acad. Sci. U.S.A. 112, E5336–E5342. 10.1073/pnas.1513447112 26351691 PMC4586888

[B20] Lo H. J. Köhler J. R. DiDomenico B. Loebenberg D. Cacciapuoti A. Fink G. R. (1997). Nonfilamentous C. albicans mutants are avirulent. Cell 90, 939–949. 10.1016/S0092-8674(00)80358-X 9298905

[B21] Lüttich A. Brunke S. Hube B. Jacobsen I. D. (2013). Serial passaging of *Candida albicans* in systemic murine infection suggests that the wild type strain SC5314 is well adapted to the murine kidney. PLoS ONE 8:e64482. 10.1371/journal.pone.0064482 23737985 PMC3667833

[B22] Marchais V. Kempf M. Licznar P. Lefrançois C. Bouchara JP. Robert R.. (2005). DNA array analysis of *Candida albicans* gene expression in response to adherence to polystyrene. FEMS Microbiology Lett. 245, 25–32. 10.1016/j.femsle.2005.02.014 15796975

[B23] Perez-Riverol Y. Csordas A. Bai J. Bernal-Llinares M. Hewapathirana S. Kundu D. J.. (2019). The PRIDE database and related tools and resources in 2019: improving support for quantification data. Nucleic Acids Res. 47, D442–D450. 10.1093/nar/gky1106 30395289 PMC6323896

[B24] Pfaller M. A. Diekema D. J. (2007). Epidemiology of invasive candidiasis: a persistent public health problem. Clin. Microbiol. Rev. 20, 133–163. 10.1128/CMR.00029-06 17223626 PMC1797637

[B25] Rashki A. Ghalehnoo Z. R. Dominguez R. (2012). The early response of *Candida albicans* filament induction is coupled with wholesale expression of the translation machinery. Comp. Clin. Path. 21, 1533–1545. 10.1007/s00580-011-1325-1

[B26] Sakita K. M. Faria D. R. Silva E. M. D. Tobaldini-Valério F. K. Kioshima E. S. Svidzinski T. I. E.. (2017). Healthcare workers' hands as a vehicle for the transmission of virulent strains of *Candida* spp.: a virulence factor approach. Microb. Pathog. 113, 225–232. 10.1016/j.micpath.2017.10.044 29074432

[B27] Si H. Hernday A. D. Hirakawa M. P. Johnson A. D. Bennett R. J. (2013). Candida albicans white and opaque cells undergo distinct programs of filamentous growth. PLoS Pathog. 9:e1003210. 10.1371/journal.ppat.1003210 23505370 PMC3591317

[B28] Thomas D. P. Bachmann S. P. Lopez-Ribot J. L. (2006). Proteomics for the analysis of the *Candida albicans* biofilm lifestyle. Proteomics 6, 5795–5904. 10.1002/pmic.200600332 17001605

[B29] Tyanova S. Temu T. Carlson A. Sinitcyn P. Mann M. Cox J. (2015). Visualization of LC-MS/MS proteomics data in MaxQuant. Proteomics 15, 1453–1456. 10.1002/pmic.201400449 25644178 PMC5024039

[B30] Tyanova S. Temu T. Sinitcyn P. Carlson A. Hein M. Y. Geiger T.. (2016). The perseus computational platform for comprehensive analysis of (prote)omics data. Nat. Methods 13, 731–740. 10.1038/nmeth.3901 27348712

[B31] Vázquez-Torres A. Balish E. (1997). Macrophages in resistance to candidiasis. Microbiol. Mol. Biol. Rev. 61, 170–192. 9184009 10.1128/mmbr.61.2.170-192.1997 PMC232606

[B32] Ying L. DeDong L. Yan W. LingCong L. Hai H. ShouTing F. (2010). Overexpression of *IFD6* in *Candida albicans* promotes biofilm formation. Acad. J. Second Military Med. Univer. 31, 599–603. 10.3724/SP.J.1008.2010.00599

