# Supplementary material for: Serial Systemic Candida albicans Infection Highlighted by Proteomics
Source: Front Cell Infect Microbiol. 2019 Jun 26;9:230. doi: 10.3389/fcimb.2019.00230 (PMC6606696; doi:10.3389/fcimb.2019.00230)
Supplement: Supplementary file 1 [file Data_Sheet_1.PDF]

Supplementary Material

Supplementary Table S1. Identification of proteins from *Candida albicans* (Passage 1) by mass spectrometry

Protein IDs	Gene name	UniProt names	Molecular function	Biological process	*Difference
**Amino acid metabolism**					
A0A1D8PG20	*ARO8*	Bifunctional 2-aminoadipate transaminase/aromatic-amino-acid:2-oxoglutarate transaminase	2-aminoadipate transaminase activity; aromatic-amino-acid:2-oxoglutarate aminotransferase activity e pyridoxal phosphate binding	aromatic amino acid family biosynthetic process; aromatic amino acid family catabolic process; aromatic amino acid family catabolic process to alcohol via Ehrlich pathway; L-lysine catabolic process; lysine biosynthetic process via aminoadipic acid	0.5215501785
A0A1D8PIB2	*ASN1*	Asparagine synthase (Glutamine-hydrolyzing) 2	asparagine synthase (glutamine-hydrolyzing) activity; ATP binding	asparagine biosynthetic process; glutamine metabolic process	0.713186264
A0A1D8PKB9	*BAT22*	Branched-chain-amino-acid aminotransferase	L-isoleucine transaminase activity; L-leucine transaminase activity; L-valine transaminase activity	branched-chain amino acid biosynthetic process	0.6488876343
Q59N40	*AAT21*	Aspartate aminotransferase	L-aspartate:2-oxoglutarate aminotransferase activity; pyridoxal phosphate binding	biosynthetic process; cellular amino acid metabolic process	-0.5720386505
Q59R18	*DED81*	Asparagine--tRNA ligase	asparagine-tRNA ligase activity; ATP binding; nucleic acid binding	asparaginyl-tRNA aminoacylation	0.819188118
Q59R20	orf19.6701	Proline--tRNA ligase	aminoacyl-tRNA editing activity; ATP binding; proline-tRNA ligase activity	prolyl-tRNA aminoacylation	0.4004220963
Q59RI1	*ILS1*	Proline--tRNA ligase	aminoacyl-tRNA editing activity; ATP binding; isoleucine-tRNA ligase activity; tRNA binding	isoleucyl-tRNA aminoacylation	0.3450279236

Q5A362	*CYS3*	Cystathionine gamma-lyase	cystathionine gamma-lyase activity; pyridoxal phosphate binding	cysteine biosynthetic process via cystathionine; methionine biosynthetic process; transsulfuration	0.8688545227
Q5AKX1	*GCV3*	Glycine cleavage system H protein	contributes_to glycine dehydrogenase (decarboxylating) activity	glycine decarboxylation via glycine cleavage system	-0.6339769363
P79023	*ARO4*	Phospho-2-dehydro-3-deoxyheptonate aldolase, tyrosine-inhibited	3-deoxy-7-phosphoheptulonate synthase activity	aromatic amino acid family biosynthetic process; chorismate biosynthetic process	0.4106874466
**Carbohydrate metabolism**					
A0A1D8PKW2	*FBP1*	Fructose 1,6-bisphosphate 1-phosphatase	fructose 1,6-bisphosphate 1-phosphatase activity	gluconeogenesis	-1.522006989
A0A1D8PLY4	*PYC2*	Pyruvate carboxylase	ATP binding; biotin binding; metal ion binding; pyruvate carboxylase activity	gluconeogenesis; pyruvate metabolic process	0.6235904694
A0A1D8PNK3	*GRE3*	Trifunctional aldehyde reductase/xylose reductase/glucose 1-dehydrogenase (NADP(+))	oxidoreductase activity	D-xylose catabolic process; arabinose catabolic process; cellular response to osmotic stress; cellular response to oxidative stress; galactose catabolic process; oxidation-reduction process	-0.9825048447
A0A1D8PS79	*IDP2*	Isocitrate dehydrogenase [NADP]	isocitrate dehydrogenase (NADP+) activity; magnesium ion binding; NAD binding	isocitrate metabolic process; tricarboxylic acid cycle	-2.973912239
A0A1D8PSH3	*CIT1*	Citrate synthase	ATP citrate synthase activity; citrate (Si)-synthase activity	tricarboxylic acid cycle	-0.8699102402
A0A1D8PSZ0	*IFE2*	Ife2p	oxidoreductase activity; zinc ion binding	oxidation-reduction process	-1.208379745
A0A1D8PU04	CAALFM_CR09670CA	S-formylglutathione hydrolase	S-formylglutathione hydrolase activity	formaldehyde catabolic process	0.6277980804
A0A1D8PU61	*FDH3*	S-(hydroxymethyl)glutathione dehydrogenase	S-(hydroxymethyl)glutathione dehydrogenase activity; zinc ion binding	cell redox homeostasis; cellular response to oxidative stress; pathogenesis; glycine catabolic process; amino acid catabolic process	0.5219736099

				to alcohol via Ehrlich pathway; ethanol oxidation; formaldehyde catabolic process; furaldehyde metabolic process; oxidation-reduction process	
P83773	*ACH1*	Acetyl-CoA hydrolase	acetyl-CoA hydrolase activity	acetate metabolic process; acetyl-CoA metabolic process; cellular response to alkaline pH	-0.7261962891
P83778	*MDH1*	Malate dehydrogenase, cytoplasmic	L-malate dehydrogenase activity; malate dehydrogenase activity	carbohydrate metabolic process; malate metabolic process; tricarboxylic acid cycle	-2.016073227
Q5A0Z9	*PDA1*	Pyruvate dehydrogenase E1 component subunit alpha	pyruvate dehydrogenase (acetyl-transferring) activity	acetyl-CoA biosynthetic process from pyruvate	0.6566324234
Q5A5V6	*PDB1*	Pyruvate dehydrogenase E1 component subunit beta	pyruvate dehydrogenase (acetyl-transferring) activity	acetyl-CoA biosynthetic process from pyruvate; glycolytic process	0.5407962799
Q5AGX8	*LAT1*	Acetyltransferase component of pyruvate dehydrogenase complex	dihydrolipoyllysine-residue acetyltransferase activity	pyruvate metabolic process	0.727148056
Q5AKV6	*PDX1*	Pdx1p	transferase activity, transferring acyl groups	filamentous growth; metabolic process; single-species biofilm formation on inanimate substrate	0.5077676773
Q5AKX2	*OSM2*	Fumarate reductase	heme binding; metal ion binding; succinate dehydrogenase activity	FAD metabolic process; oxidation-reduction process; protein folding in endoplasmic reticulum	0.7646503448
Q5AKX8	*CYB2*	Cyb2p	heme binding; L-lactate dehydrogenase (cytochrome) activity; metal ion binding	entry into host through natural portals; lactate metabolic process; metabolism by symbiont of substance in host	0.5137434006
**Lipid metabolism**					
A0A1D8PH78	*ERG20*	Bifunctional (2E,6E)-farnesyl diphosphate synthase/dimethylallyltransferase	dimethylallyltranstransferase activity; geranyltranstransferase activity	ergosterol biosynthetic process; isoprenoid biosynthetic process	0.9012012482
**Protein biosynthesis/folding**					

A0A1D8PN90	*STI1*	Hsp90 cochaperone	ATPase inhibitor activity; Hsp70 protein binding; Hsp90 protein binding; mRNA binding	protein folding; protein targeting to mitochondrion	-0.4388208389
P25997	*CEF3*	Elongation factor 3	ATPase activity; ATP binding; GTPase activity; translation elongation factor activity	translational elongation	0.4013900757
**Glyoxylate cycle**					
Q5APD2	*MLS1*	Malate synthase	malate synthase activity	glyoxylate cycle; tricarboxylic acid cycle	-2.737627983
**Stress response**					
A0A1D8PLJ3	*SOD1*	Superoxide dismutase [Cu-Zn]	metal ion binding; superoxide dismutase activity	cellular response to oxidative stress; filamentous growth; filamentous growth of a population of unicellular organisms in response to starvation; pathogenesis	-2.281773567
A0A1D8PQH5	*SOD3*	Superoxide dismutase	metal ion binding; superoxide dismutase activity	age-dependent response to oxidative stress involved in chronological cell aging; oxidation-reduction process; removal of superoxide radicals	1.609313965
A0A1D8PS56	*ECM4*	Omega-class glutathione transferase	glutathione transferase activity	cellular response to starvation; filamentous growth; filamentous growth of a population of unicellular organisms in response to biotic stimulus; filamentous growth of a population of unicellular organisms in response to starvation	-0.2444143295
O13289	*CAT1*	Peroxisomal catalase	catalase activity; heme binding; metal ion binding	cellular response to hydrogen peroxide; cellular response to starvation; filamentous growth; filamentous growth of a population of unicellular organisms in response to chemical stimulus; filamentous growth of a population of unicellular organisms in response to starvation; hydrogen peroxide catabolic process; hydrogen peroxide metabolic process; interaction with host; pathogenesis; response to hydrogen peroxide	-0.3804578781

Q59MV9	*YHB1*	Flavohemoprotein	heme binding; metal ion binding; nitric oxide dioxygenase activity; oxygen binding	cellular response to nitrosative stress; filamentous growth; filamentous growth of a population of unicellular organisms; nitric oxide catabolic process; pathogenesis; response to defense-related host nitric oxide production; response to toxic substance	0.6299734116
Q59WW7	*GPS2/* orf19.86	Glutathione peroxidase	glutathione peroxidase activity	response to oxidative stress	-0.959485054
Q5A5A0	*PRX1*	Thioredoxin peroxidase	thioredoxin peroxidase activity	cell redox homeostasis; cellular response to oxidative stress	-0.4752588272
Q5ABB1	*TTR1*	Dithiol glutaredoxin	electron transfer activity; protein disulfide oxidoreductase activity	cell redox homeostasis; cellular response to oxidative stress; pathogenesis	-0.4375391006
**Ribosome**					
A0A1D8PDL6	orf19.2478.1	Ribosomal 60S subunit protein L7A	cellular component	cytoplasmic translation (IEA with S. cerevisiae: RPL7A) e maturation of LSU-rRNA	0.5493927002
A0A1D8PDP4	*RPS27A*	40S ribosomal protein S27	metal ion binding e structural constituent of ribosome	translation	-0.8073043823
A0A1D8PU46	*SIK1*	snoRNP complex protein	unknown	rRNA processing	0.2416696548
Q59S06	*NOP58*	Nucleolar protein 58	unknown	filamentous growth; rRNA processing	0.3969116211
Q5A0V9	*NOP1*	rRNA methyltransferase	methyltransferase activity; RNA binding	rRNA processing	0.4075584412
**Metabolism of cofactors and vitamins**					
Q5A3V6	*RIB3*	3,4-dihydroxy-2-butanone 4-phosphate synthase	3,4-dihydroxy-2-butanone-4-phosphate synthase activity; metal ion binding	riboflavin biosynthetic process	0.7043361664
Q5A3Y5	*THI13*	4-amino-5-hydroxymethyl-2-methylpyrimidine phosphate synthase	thiamine pyrophosphate binding	thiamine biosynthetic process; thiamine diphosphate biosynthetic process	1.331450462

Q5ANB7	*THI4*	Thiamine thiazole synthase	metal ion binding	response to stress; thiamine biosynthetic process	1.799358368
**Energy**					
A0A1D8PJ01	*PMA1*	Plasma membrane ATPase	ATPase activity; ATP binding; metal ion binding; proton-exporting ATPase activity, phosphorylative mechanism	proton export across plasma membrane	0.7011356354
Q5AP79	*MIR1*	Mir1p	phosphate ion transmembrane transporter activity	phosphate ion transport	0.4497089386
Q59ZE0	*ATP4*	F1F0 ATP synthase subunit 4	proton transmembrane transporter activity	ATP synthesis coupled proton transport	0.225028038
**Others**					
A0A1D8PD78	*IFD6*	Ifd6p	oxidoreductase activity e aryl-alcohol dehydrogenase (NAD+) activity	single-species biofilm formation on inanimate substrate	1.287586212
A0A1D8PDA4	*FMA1*	Fma1p	oxidoreductase activity	metabolic process	0.8170814514
A0A1D8PHQ3	orf19.36.1	Uncharacterized protein	unknown	unknown	-0.6240568161
O13318	*PHR2*	pH-responsive protein 2	1,3-beta-glucanosyltransferase activity; glucanosyltransferase activity	fungal-type cell wall organization; pathogenesis	0.7462463379
Q59KV8	*LSP1*	Lipid-binding protein	lipid binding	eisosome assembly; endocytosis; negative regulation of protein kinase activity; negative regulation of sphingolipid biosynthetic process; protein localization to eisosome filament; response to heat	-0.4921894073
Q59Y31	*YWP1*	Yeast-form wall Protein 1	unknown	adhesion of symbiont to host; cell adhesion; single-species biofilm formation	0.5545415878
Q5AK88	orf19.3932	Uncharacterized protein	nucleic acid binding	unknown	-0.3563690186

*Difference of the protein intensities between P1 and wild type strain, positive value indicating increased in abundance in P1 and negative value indicating decreased in abundance in P1.

Molecular function and biological process according to UniProt and *Candida* genome database.

Supplementary Table 2. Identification of proteins from *Candida albicans* (Passage 3) by mass spectrometry

Protein IDs	Gene name	UniProt names	Molecular function	Biological process	*Difference
**Amino acid metabolism**					
A0A1D8PGT5	*ALD5*	Aldehyde dehydrogenase (NAD(P)(+))	oxidoreductase activity, acting on the aldehyde or oxo group of donors, NAD or NADP as acceptor	acetate biosynthetic process; oxidation-reduction process	-0.7793941498
A0A1D8PMH8	*GDH3*	Glutamate dehydrogenase	oxidoreductase activity, acting on the CH-NH2 group of donors, NAD or NADP as acceptor	cellular amino acid metabolic process	-0.6970434189
Q5A362	*CYS3*	Cystathionine gamma-lyase	cystathionine gamma-lyase activity; pyridoxal phosphate binding	cysteine biosynthetic process via cystathionine; methionine biosynthetic process; transsulfuration	0.681183815
**Carbohydrate metabolism**					
A0A1D8PKV4	*FUM12*	Fum12p	fumarate hydratase activity	fumarate metabolic process; tricarboxylic acid cycle	-0.4057559967
A0A1D8PKW2	*FBP1*	Fructose 1,6-bisphosphate 1-phosphatase	fructose 1,6-bisphosphate 1-phosphatase activity	gluconeogenesis	-1.168065071
A0A1D8PSH3	*CIT1*	Citrate synthase	ATP citrate synthase activity; citrate (Si)-synthase activity	tricarboxylic acid cycle	-1.065693855
A0A1D8PSZ0	*IFE2*	Ife2p	oxidoreductase activity; zinc ion binding	oxidation-reduction process	-1.049014091
P83773	*ACH1*	Acetyl-CoA hydrolase	acetyl-CoA hydrolase activity	acetate metabolic process; acetyl-CoA metabolic process; cellular response to alkaline pH	-0.6624755859
Q5AED0	orf19.338	Uncharacterized protein	mannosyl-oligosaccharide glucosidase activity	oligosaccharide metabolic process	-0.4185562134
Q8NJN3	*ACS2*	Acetyl-coenzyme A synthetase 2	acetate-CoA ligase activity; AMP binding; ATP binding	acetyl-CoA biosynthetic process; acetyl-CoA biosynthetic process from acetate	-0.4514570236
**Lipid metabolism**					

A0A1D8PRR7	*ACC1*	Acetyl-CoA carboxylase	acetyl-CoA carboxylase activity; ATP binding; metal ion binding	fatty acid biosynthetic process	-0.3735637665
**Nucleic acid processing**					
Q59VN4	*HHF22/ HHF1*	Histone H4	DNA binding; protein heterodimerization activity	DNA-templated transcription, initiation; nucleosome assembly	0.4918985367
Q5AI86	*TIF34*	Eukaryotic translation initiation factor 3 subunit I	translation initiation factor activity	cellular response to drug	0.461016655
**Protein biosynthesis/folding**					
A0A1D8PQ94	*SBA1*	Hsp90 cochaperone	chaperone binding	protein folding	0.7337417603
**Glyoxylate cycle**					
Q59RB8	*ICL1*	Isocitrate lyase	isocitrate lyase activity; metal ion binding	glyoxylate cycle; pathogenesis	-3.102285385
Q5APD2	*MLS1*	Malate synthase	malate synthase activity	glyoxylate cycle; tricarboxylic acid cycle	-3.049711227
**Stress response**					
A0A1D8PLJ3	*SOD1*	Superoxide dismutase [Cu-Zn]	metal ion binding; superoxide dismutase activity	cellular response to oxidative stress; filamentous growth; filamentous growth of a population of unicellular organisms in response to starvation; pathogenesis	-2.582626343
A0A1D8PQH5	*SOD3*	Superoxide dismutase	metal ion binding; superoxide dismutase activity	age-dependent response to oxidative stress involved in chronological cell aging; oxidation-reduction process; removal of superoxide radicals	1.941585541
O13289	*CAT1*	Peroxisomal catalase	catalase activity; heme binding; metal ion binding	cellular response to hydrogen peroxide; cellular response to starvation; filamentous growth; filamentous growth of a population of unicellular organisms in response to chemical stimulus; filamentous growth of a population of unicellular organisms in response to starvation; hydrogen peroxide catabolic process; hydrogen peroxide metabolic process;	-0.4710092545

				interaction with host; pathogenesis; response to hydrogen peroxide	
Q59MV9	*YHB1*	Flavohemoprotein	heme binding; metal ion binding; nitric oxide dioxygenase activity; oxygen binding	cellular response to nitrosative stress; filamentous growth; filamentous growth of a population of unicellular organisms; nitric oxide catabolic process; pathogenesis; response to defense-related host nitric oxide production; response to toxic substance	0.5347242355
**Metabolism of cofactors and vitamins**					
Q5A3V6	*RIB3*	3,4-dihydroxy-2-butanone 4-phosphate synthase	3,4-dihydroxy-2-butanone-4-phosphate synthase activity; metal ion binding	riboflavin biosynthetic process	0.4435052872
Q5A3Y5	*THI13*	4-amino-5-hydroxymethyl-2-methylpyrimidine phosphate synthase	thiamine pyrophosphate binding	thiamine biosynthetic process; thiamine diphosphate biosynthetic process	0.8529376984
**Energy metabolism**					
Q9B8D8	*COX2*	Cytochrome c oxidase subunit 2	copper ion binding; cytochrome-c oxidase activity	mitochondrial electron transport, cytochrome c to oxygen	-0.6721410751
**Others**					
A0A1D8PHF8	*WH11*	Wh11p	unknown	pathogenesis; phenotypic switching; response to stress; single-species biofilm formation on inanimate substrate	-0.8011341095
A0A1D8PHH2	*PNG2*	Png2p	peptide-N4-(N-acetyl-beta-glucosaminyl)asparagine amidase activity	protein deglycosylation	0.8540554047
A0A1D8PM81	orf19.3782.2	MICOS complex subunit MIC10	unknown	cristae formation/ protein transport	-1.16242218
A0A1D8PRB4	orf19.1338	Uncharacterized protein	unknown	unknown	0.3890743256
A0A1D8PSE1	*MLC1*	Mlc1p	calcium ion binding	mitotic actomyosin contractile ring assembly actin filament organization; protein localization to medial cortex; vesicle targeting	0.3875980377

Q59Y31	*YWP1*	Yeast-form wall Protein 1	unknown	adhesion of symbiont to host; cell adhesion; single-species biofilm formation	1.27693367

*Difference of the protein intensities between P3 and wild type strain, positive value indicating increased in abundance in P3 and negative value indicating decreased in abundance in P3.

Molecular function and biological process according to UniProt and *Candida* genome database.

Supplementary Table 3. Identification of proteins from *Candida albicans* (Passage 4) by mass spectrometry

Protein IDs	Gene name	UniProt names	Molecular function	Biological process	*Difference
**Amino acid metabolism**					
A0A1D8PDX5	*MMD1*	Isoleucine biosynthesis protein	deaminase activity	isoleucine biosynthetic process; mitochondrial translation	-0.4822034836
A0A1D8PF68	*SAM2*	S-adenosylmethionine synthase	ATP binding; metal ion binding; methionine adenosyltransferase activity	one-carbon metabolic process; S-adenosylmethionine biosynthetic process	0.6287565231
A0A1D8PGT5	*ALD5*	Aldehyde dehydrogenase (NAD(P)(+))	oxidoreductase activity, acting on the aldehyde or oxo group of donors, NAD or NADP as acceptor	acetate biosynthetic process; oxidation-reduction process	-0.6962976456
A0A1D8PIB2	*ASN1*	Asparagine synthase (Glutamine-hydrolyzing) 2	asparagine synthase (glutamine-hydrolyzing) activity; ATP binding	asparagine biosynthetic process; glutamine metabolic process	0.2279081345
A0A1D8PJR2	*GTT11*	Gtt11p	glutathione transferase activity	unknown	-0.580198288
A0A1D8PRR5	*ARG1*	argininosuccinate synthase	argininosuccinate synthase activity; ATP binding	arginine biosynthetic process	-0.7058420181
P83783	*SAH1*	Adenosylhomocysteinase	adenosylhomocysteinase activity; NAD binding	one-carbon metabolic process; S-adenosylhomocysteine catabolic process	0.4408073425
Q59MU3	*ARO10*	Phenylpyruvate decarboxylase	carboxy-lyase activity; metal ion binding; thiamine pyrophosphate binding; phenylpyruvate decarboxylase activity	aromatic amino acid family catabolic process to alcohol via Ehrlich pathway	-0.4963207245
Q59N40	*AAT21*	Aspartate aminotransferase	L-aspartate:2-oxoglutarate aminotransferase activity; pyridoxal phosphate binding	biosynthetic process; cellular amino acid metabolic process	-0.5414867401
Q59R18	*DED81*	Asparagine--tRNA ligase	asparagine-tRNA ligase activity; ATP binding; nucleic acid binding	asparaginyl-tRNA aminoacylation	0.5847959518
Q59RI1	*ILS1*	Isoleucine--tRNA ligase	aminoacyl-tRNA editing activity; ATP binding; isoleucine-tRNA ligase activity; tRNA binding	isoleucyl-tRNA aminoacylation	-0.3195981979

Q5A362	*CYS3*	Cystathionine gamma-lyase	lyase activity; pyridoxal phosphate binding	cysteine biosynthetic process via cystathionine; methionine biosynthetic process; transsulfuration	0.9481925964
**Carbohydrate metabolism**					
A0A1D8PKV4	*FUM12*	Fum12p	fumarate hydratase activity	fumarate metabolic process; tricarboxylic acid cycle	-0.724240303
A0A1D8PKW2	*FBP1*	Fructose 1,6-bisphosphate 1-phosphatase	fructose 1,6-bisphosphate 1-phosphatase activity	gluconeogenesis	-1.452363968
A0A1D8PLY4	*PYC2*	Pyruvate carboxylase	ATP binding; biotin binding; metal ion binding; pyruvate carboxylase activity	gluconeogenesis; pyruvate metabolic process	0.3502264023
A0A1D8PNK3	*GRE3*	Trifunctional aldehyde reductase/xylose reductase/glucose 1-dehydrogenase (NADP(+))	oxidoreductase activity	D-xylose catabolic process; arabinose catabolic process; cellular response to osmotic stress; cellular response to oxidative stress; galactose catabolic process; oxidation-reduction process	-0.9244403839
A0A1D8PP43	*ADH1*	Adh1p	alcohol dehydrogenase (NAD) activity; methylglyoxal reductase (NADH-dependent) activity; zinc ion binding	induction by symbiont of host defense response; interaction with host; single-species biofilm formation in or on host organism; single-species biofilm formation on inanimate substrate	0.8220968246
A0A1D8PS79	*IDP2*	Isocitrate dehydrogenase [NADP]	isocitrate dehydrogenase (NADP+) activity; magnesium ion binding; NAD binding	isocitrate metabolic process; tricarboxylic acid cycle	-2.27148819
A0A1D8PSA9	*PGM2*	phosphoglucomutase	intramolecular transferase activity, phosphotransferases; magnesium ion binding	carbohydrate metabolic process	-0.3903055191
A0A1D8PSH3	*CIT1*	Citrate synthase	ATP citrate synthase activity; citrate (Si)-synthase activity	tricarboxylic acid cycle	-1.223953247
A0A1D8PSZ0	*IFE2*	Ife2p	oxidoreductase activity; zinc ion binding	oxidation-reduction process	-1.499850273

A0A1D8PUB4	*XYL2*	L-iditol 2-dehydrogenase	oxidoreductase activity; zinc ion binding	oxidation-reduction process	-0.3436594009
O93827	*MPG1/SRB1*	Mannose-1-phosphate guanyltransferase	GTP binding; mannose-1-phosphate guanylyltransferase activity	cell cycle; cell wall mannoprotein biosynthetic process; GDP-mannose biosynthetic process	0.3982839584
P40953	*CHT2*	Chitinase 2	chitinase activity; chitin binding	cellular response to starvation; chitin catabolic process; filamentous growth of a population of unicellular organisms in response to starvation; polysaccharide catabolic process; carbohydrate metabolic process	-0.5086317062
P46614	*CDC19*	Pyruvate kinase	ATP binding; kinase activity; magnesium ion binding; potassium ion binding; pyruvate kinase activity	cellular response to starvation; filamentous growth; filamentous growth of a population of unicellular organisms in response to biotic stimulus; filamentous growth of a population of unicellular organisms in response to starvation; glycolytic process; induction by symbiont of host defense response	0.5644741058
P83773	*ACH1*	Acetyl-CoA hydrolase	acetyl-CoA hydrolase activity	acetate metabolic process; acetyl-CoA metabolic process; cellular response to alkaline pH	-1.031401634
P83778	*MDH1*	Malate dehydrogenase, cytoplasmic	L-malate dehydrogenase activity; malate dehydrogenase activity	carbohydrate metabolic process; malate metabolic process; tricarboxylic acid cycle; gluconeogenesis	-1.569755554
P83779	*PDC11*	Pyruvate decarboxylase	magnesium ion binding; pyruvate decarboxylase activity; thiamine pyrophosphate binding	L-phenylalanine catabolic process; aromatic amino acid family catabolic process to alcohol via Ehrlich pathway; glycolytic fermentation to ethanol; tryptophan catabolic process	0.4423465729
Q5A850	*GSY1*	Glycogen [starch] synthase	glycogen (starch) synthase activity	glycogen biosynthetic process	-0.2303543091
Q5AGZ8	*PFK2*	ATP-dependent 6-phosphofructokinase	6-phosphofructokinase activity; ATP binding; metal ion binding	fructose 6-phosphate metabolic process; glycolytic process	0.3181686401
Q5AKX2	*OSM2*	Fumarate reductase	heme binding; metal ion binding; succinate dehydrogenase activity	FAD metabolic process; oxidation-reduction process; protein folding in endoplasmic reticulum	0.5936632156

Q5AKX8	*CYB2*	Cyb2p	heme binding; L-lactate dehydrogenase (cytochrome) activity; metal ion binding	entry into host through natural portals; lactate metabolic process; metabolism by symbiont of substance in host	0.3186035156
Q5AMP4	*MDH1-1*	Malate dehydrogenase	L-malate dehydrogenase activity	carbohydrate metabolic process; malate metabolic process; tricarboxylic acid cycle	-0.5831956863
Q8NJN3	*ACS2*	Acetyl-coenzyme A synthetase 2	acetate-CoA ligase activity; AMP binding; ATP binding	acetyl-CoA biosynthetic process; acetyl-CoA biosynthetic process from acetate	-0.4534273148
**Lipid metabolism**					
A0A1D8PQN3	*ACB1*	Long-chain fatty acid transporter	long-chain fatty acyl-CoA binding	chronological cell aging; very long-chain fatty acid biosynthetic process	1.179416656
A0A1D8PQP7	*CYB5*	Cyb5p	electron transfer activity; heme binding; metal ion binding	ergosterol biosynthetic process	0.4321460724
A0A1D8PH52	*ERG10*	Acetyl-CoA C-acetyltransferase	transferase activity, transferring acyl groups other than amino-acyl groups	metabolic process	-0.5487518311
A0A1D8PRR7	*ACC1*	Acetyl-CoA carboxylase	acetyl-CoA carboxylase activity; ATP binding; metal ion binding	fatty acid biosynthetic process	-0.4630470276
Q5A7M9	*RHR2*	Glycerol-1-phosphatase	glycerol-3-phosphatase activity; phosphatase activity	cell-abiotic substrate adhesion; cellular response to osmotic stress; entry into host; glycerol biosynthetic process; glycerol metabolic process; pathogenesis; single-species biofilm formation on inanimate substrate	0.721950531
**Nucleic acid processing**					
A0A1D8PFX4	orf19.7256	RNA-binding protein	contributes_to RNA binding; poly(U) RNA binding	mRNA splicing, via spliceosome	1.159379005
A0A1D8PH31	*TAF14*	TATA-binding protein-associated factor	DNA-binding transcription factor activity; contributes_to DNA translocase activity; TBP-class protein binding; transcription factor activity, core RNA polymerase II binding	positive regulation of cell adhesion involved in single-species biofilm formation; positive regulation of cell-substrate adhesion; regulation of single-species biofilm formation on inanimate substrate; regulation of transcription by RNA polymerase II; single-	-0.2888507843

				species biofilm formation on inanimate substrate	
Q59WG0	*HNT1*	Adenosine 5'-monophosphoramidase	catalytic activity	nucleotide metabolic process	-1.065743446
Q5AG96	orf19.4283	Uncharacterized protein	translation initiation factor activity	cytoplasmic translational initiation	-0.5718517303
Q9P975	*TIF45*	Eukaryotic translation initiation factor 4E	translation initiation factor activity	regulation of translation; translational initiation	-0.6807031631
**Protein biosynthesis/folding**					
A0A1D8PFU8	orf19.7215.3	Uncharacterized protein	chaperone binding; unfolded protein binding	protein folding; chaperone-mediated protein complex assembly; protein import into mitochondrial intermembrane space	-0.8519191742
A0A1D8PJ20	*SCL1*	Proteasome endopeptidase complex	threonine-type endopeptidase activity	ubiquitin-dependent protein catabolic process	-0.8875684738
A0A1D8PM35	*EFB1*	Translation elongation factor 1 subunit beta	translation elongation factor activity	translational elongation	-0.3518152237
A0A1D8PRB6	*PUP3*	proteasome core particle subunit beta 3	threonine-type endopeptidase activity	proteasome-mediated ubiquitin-dependent protein catabolic process	-0.4264621735
A0A1D8PRC2	*CPY1*	carboxypeptidase	serine-type carboxypeptidase activity	macroautophagy; phytochelatin biosynthetic process; zymogen activation	0.6612825394
O94083	*ANB1*	Eukaryotic translation initiation factor 5A	ribosome binding; translation elongation factor activity	positive regulation of translational elongation; positive regulation of translational termination; translational frameshifting	-0.4636182785
Q5A0L8	*PR26*	Proteasome regulatory particle base subunit	ATP binding; proteasome-activating ATPase activity; hydrolase activity; nucleoside-triphosphatase activity; nucleotide binding	protein catabolic process	-0.4392995834
Q5ALM6	*CPR3*	Peptidyl-prolyl cis-trans isomerase	peptidyl-prolyl cis-trans isomerase activity	protein folding; apoptotic process; protein peptidyl-prolyl isomerization	-0.6788568497
Q59WE2	*SKP1*	SCF ubiquitin ligase subunit	ligase activity	ubiquitin-dependent protein catabolic process	0.5514116287
**Glyoxylate cycle**					

Q59RB8	*ICL1*	Isocitrate lyase	isocitrate lyase activity; metal ion binding	glyoxylate cycle; pathogenesis	-4.157952309
Q5APD2	*MLS1*	Malate synthase	malate synthase activity	glyoxylate cycle; tricarboxylic acid cycle	-3.086671829
**Stress response**					
A0A1D8PLJ3	*SOD1*	Superoxide dismutase [Cu-Zn]	metal ion binding; superoxide dismutase activity	cellular response to oxidative stress; filamentous growth; filamentous growth of a population of unicellular organisms in response to starvation; pathogenesis	-1.731225967
A0A1D8PMP0	*OYE32*	Oye32p	FMN binding; oxidoreductase activity	cell redox homeostasis	0.6551866531
A0A1D8PQH5	*SOD3*	Superoxide dismutase	metal ion binding; superoxide dismutase activity	age-dependent response to oxidative stress involved in chronological cell aging; oxidation-reduction process; removal of superoxide radicals	2.057108879
A0A1D8PS56	*ECM4*	Omega-class glutathione transferase	glutathione transferase activity	cellular response to starvation; filamentous growth; filamentous growth of a population of unicellular organisms in response to biotic stimulus; filamentous growth of a population of unicellular organisms in response to starvation	-0.4098491669
A0A1D8PSE7	*IFR2*	Ifr2p	oxidoreductase activity	oxidation-reduction process	-0.5328969955
A0A1D8PTP9	*HSP104*	chaperone ATPase	ATPase activity, coupled; ATP binding	cellular heat acclimation; cellular response to heat; chaperone cofactor-dependent protein refolding; pathogenesis; protein metabolic process; single-species biofilm formation on inanimate substrate	-0.2231674194
O13289	*CAT1*	Peroxisomal catalase	catalase activity; heme binding; metal ion binding	cellular response to hydrogen peroxide; cellular response to starvation; filamentous growth; filamentous growth of a population of unicellular organisms in response to chemical stimulus; filamentous growth of a population of unicellular organisms in response to starvation; hydrogen peroxide catabolic process; hydrogen peroxide metabolic process;	-0.3214054108

				interaction with host; pathogenesis; response to hydrogen peroxide	
O74261	*HSP60*	Heat shock protein 60, mitochondrial	ATP binding	cellular response to heat; protein refolding	-0.2773160934
P0CT51	*BLP1*	Blood-induced peptide 1	unknown	unknown	1.216862679
Q59MV9	*YHB1*	Flavohemoprotein	heme binding; metal ion binding; nitric oxide dioxygenase activity; oxygen binding	cellular response to nitrosative stress; filamentous growth; filamentous growth of a population of unicellular organisms; nitric oxide catabolic process; pathogenesis; response to defense-related host nitric oxide production; response to toxic substance	0.3077068329
Q59X49	*DDR48*	Stress protein DDR48	ATPase activity; GTPase activity	cellular response to oxidative stress; cellular response to starvation; DNA repair; filamentous growth; filamentous growth of a population of unicellular organisms in response to biotic stimulus and to starvation	1.294729233
Q5A7P9	*DOT5*	Thioredoxin peroxidase	peroxidase activity	cell redox homeostasis	-0.483587265
Q5AEN1	*CCP1*	Cytochrome c peroxidase, mitochondrial	cytochrome-c peroxidase activity; heme binding; metal ion binding	cellular response to oxidative stress; cellular response to reactive oxygen species	0.3090362549
**Mitochondrial biogenesis**					
A0A1D8PM81	orf19.3782.2	MICOS complex subunit MIC10	unknown	cristae formation	-1.53439045
Q59R24	*TIM9*	Mitochondrial import inner membrane translocase subunit TIM9	metal ion binding	protein transport	0.7146520615
Q5AH14	*TOM40*	Tom40p	protein transmembrane transporter activity	protein import into mitochondrial matrix	-0.4477071762
Q5AND0	*PHB2*	Prohibitin subunit	unknown	mitochondrion inheritance; negative regulation of proteolysis; protein folding; replicative cell aging	-0.3015499115
**Ribosome**					

A0A1D8PTS0	*RPP2A*	ribosomal protein P2A	structural constituent of ribosome	translation; translational elongation	0.7037200928
A0A1D8PU46	*SIK1*	snoRNP complex protein	unknown	rRNA processing	-0.2836961746
Q5A109;Q5AD S0	*UBI3;UBI4*	Ubiquitin-ribosomal 40S subunit protein S31 fusion protein/ Ubiquitin	protein tag; structural constituent of ribosome/ protein tag	protein ubiquitination; ribosome biogenesis; translation/ cell morphogenesis; cellular response to heat; filamentous growth; filamentous growth of a population of unicellular organisms; pathogenesis; phenotypic switching; protein ubiquitination	0.6953516006
Q5AGZ7	*RPL5*	Ribosomal 60S subunit protein L5	5S rRNA binding; structural constituent of ribosome	translation	-0.4783248901
**Metabolism of cofactors and vitamins**					
A0A1D8PHR5	*PST1*	Pst1p	FMN binding; NAD(P)H dehydrogenase (quinone) activity; oxidoreductase activity, acting on NAD(P)H, quinone or similar compound as acceptor	cellular response to oxidative stress; pathogenesis ; negative regulation of transcription, DNA-templated	-1.439580917
Q5A3V6	*RIB3*	3,4-dihydroxy-2-butanone 4-phosphate synthase	3,4-dihydroxy-2-butanone-4-phosphate synthase activity; metal ion binding	riboflavin biosynthetic process	0.6501731873
Q5A3Y5	*THI13*	4-amino-5-hydroxymethyl-2-methylpyrimidine phosphate synthase	thiamine pyrophosphate binding	thiamine biosynthetic process; thiamine diphosphate biosynthetic process;	0.9725437164
Q5ANB7	*THI4*	Thiamine thiazole synthase	metal ion binding	response to stress; thiamine biosynthetic process	0.7297010422
Q59Y37	*PST2*	Pst2p	2-hydroxy-1,4-benzoquinone reductase activity; FMN binding; NAD(P)H dehydrogenase (quinone) activity	cellular response to oxidative stress; pathogenesis; negative regulation of transcription, DNA-templated	-0.5678453445
**Energy & respiration**					

A0A1D8PDP8	orf19.2439.1	Ubiquinol--cytochrome-c reductase subunit 10	contributes_to ubiquinol-cytochrome-c reductase activity	aerobic respiration; mitochondrial electron transport, ubiquinol to cytochrome c	-0.8264064789
A0A1D8PQD5	orf19.1082.1	cytochrome c oxidase subunit	cytochrome-c oxidase activity	mitochondrial intermembrane space; mitochondrial respiratory chain complex IV	0.5557098389
Q59KG2	*RGI1/UCF1*	Respiratory growth induced protein 1	unknown	energy reserve metabolic process	-0.7740736008
**Others**					
A0A1D8PHH2	*PNG2*	Png2p	peptide-N4-(N-acetyl-beta-glucosaminyl)asparagine amidase activity	protein deglycosylation	2.096619606
A0A1D8PHQ3	orf19.36.1	Uncharacterized protein	unknown	unknown	1.899688721
A0A1D8PHU6	orf19.2269	Putative phosphoric monoester hydrolase	phosphatase activity	metabolic process	-0.6465187073
A0A1D8PLD7	orf19.4609	Uncharacterized protein	hydrolase activity	unknown	-0.4570531845
A0A1D8PMF8	*CMD1*	Calmodulin	calcium-dependent protein binding; calcium ion binding	cellular response to drug; filamentous growth of a population of unicellular organisms; intracellular signal transduction; phospholipid metabolic process	0.5214548111
A0A1D8PQE6	orf19.2125	Uncharacterized protein	unknown	unknown	0.9611930847
A0A1D8PTR7	*TPM2*	Tropomyosin	actin lateral binding	actin cortical patch localization; actin cortical patch organization; actin filament bundle assembly; actin filament reorganization; mitotic actomyosin contractile ring assembly; mitotic actomyosin contractile ring contraction; ...; pseudohyphal growth	0.7659692764
G1UAZ9	orf19.5158	Uncharacterized protein	oxidoreductase activity	oxidation-reduction process	-0.59416008
O13318	*PHR2*	pH-responsive protein 2	1,3-beta-glucanosyltransferase activity; glucanosyltransferase activity	fungal-type cell wall organization; pathogenesis	1.279542923

Q59Y31	*YWP1*	Yeast-form wall Protein 1	unknown	adhesion of symbiont to host; cell adhesion; single-species biofilm formation	1.614230156
Q5A1M1	*TFS1*	Tfs1p	peptidase inhibitor activity; phospholipid binding	regulation of Ras protein signal transduction; regulation of proteolysis	-0.6290254593
Q5AD47;A0A1 D8PG82	*HGT6;HGT8*	Hexose transporter/Hgt8p	transmembrane transporter activity	carbohydrate transport; glucose transmembrane transport	0.5088605881
Q5AF37	orf19.2769	Uncharacterized protein	Unknown	Unknown	1.010421753

*Difference of the protein intensities between P4 and wild type strain, positive value indicating increased in abundance in P4 and negative value indicating decreased in abundance in P4.

Molecular function and biological process according to UniProt and *Candida* genome database.
